# A Dielectric Elastomer Actuator-Driven Vibro-Impact Crawling Robot

**DOI:** 10.3390/mi13101660

**Published:** 2022-10-02

**Authors:** Chuang Wu, Huan Yan, Anjiang Cai, Chongjing Cao

**Affiliations:** 1 School of Mechanical and Electrical Engineering, Xi’an University of Architecture and Technology, Xi’an 710055, China; 2 Research Centre for Medical Robotics and Minimally Invasive Surgical Devices, Shenzhen Institute of Advanced Technology (SIAT), Chinese Academy of Sciences, Shenzhen 518055, China

**Keywords:** dielectric elastomer actuator (DEA), soft robot, vibro-impact, crawling robot

## Abstract

Over the last decade, many bio-inspired crawling robots have been proposed by adopting the principle of two-anchor crawling or anisotropic friction-based vibrational crawling. However, these robots are complicated in structure and vulnerable to contamination, which seriously limits their practical application. Therefore, a novel vibro-impact crawling robot driven by a dielectric elastomer actuator (DEA) is proposed in this paper, which attempts to address the limitations of the existing crawling robots. The novelty of the proposed vibro-impact robot lies in the elimination of anchoring mechanisms or tilted bristles in conventional crawling robots, hence reducing the complexity of manufacturing and improving adaptability. A comprehensive experimental approach was adopted to characterize the performance of the robot. First, the dynamic response of the DEA-impact constraint system was characterized in experiments. Second, the performance of the robot was extensively studied and the fundamental mechanisms of the vibro-impact crawling locomotion were analyzed. In addition, effects of several key parameters on the robot’s velocity were investigated. It is demonstrated that our robot can realize bidirectional motion (both forward and backward) by simple tuning of the key control parameters. The robot demonstrates a maximum forward velocity of 21.4 mm/s (equivalent to 0.71 body-length/s), a backward velocity of 16.9 mm/s, and a load carrying capacity of 9.5 g (equivalent to its own weight). The outcomes of this paper can offer guidelines for high-performance crawling robot designs, and have potential applications in industrial pipeline inspections, capsule endoscopes, and disaster rescues.

## 1. Introduction

In nature, many organisms move through various environments using a crawling gait, such as earthworms [1], inchworms [2], and leeches [3]. Inspired by this, a variety of crawling robots have been developed in recent years. These robots have broad prospects in various practical applications [4,5,6,7,8,9], such as capsule endoscopes, engineering diagnosis, and disaster rescue.

The movement of most crawling robots is realized utilizing the principle of two-anchor crawling, or anisotropic friction-based vibrational crawling. The principle of two-anchor crawling mimics the inchworm mechanism, which mainly uses the orderly attachment and release of the front and rear anchors as well as the elongation and contraction of the main body to achieve directional locomotion. Moreira et al. [10] proposed a 3D printed three-part inchworm robot with two driving servomotors and passive friction pads. The inchworm robot can reach a velocity of 2.54 cm/s and crawl on an inclined plane of 19°. Shi et al. [11] developed a crawling robot driven by shape memory alloy (SMA) wire to imitate the inchworm’s abdominal contraction; a ratchet structure was used to mimic the inchworm’s feet. Chang et al. [12] proposed a flexible sheet-based inchworm robot with two different locomotion modes. It can crawl upstairs in an ‘S’ shape mode and pass through low gaps in an omega-shaped locomotion mode. Zhou et al. [13] designed a millimeter-scale ground crawling robot driven by a novel prismatic mechanism capable of operating in a wide range of actuation frequencies, achieving a maximum ground crawling velocity of ~24 body-length(BL)/s. Muralidharan et al. [14] developed a biomimetic soft worm robot driven by SMA which utilized the bending behavior of the robot to achieve steering in peristaltic and two-anchor locomotion. Duduta et al. [15] proposed a soft robot driven by DEA and explored a range of actuation modes combined with a novel actuator design to enable multi-modal locomotion, including crawling, hopping, jumping, and rolling. Verma et al. [16] demonstrated a pneumatic soft robot based on a buckling pneumatic actuator that is capable of crawling in tubes with turns, inclines, and variable-diameters. Wang et al. [17] proposed a novel expansion mechanism for a gastrointestinal microrobot by adopting a double-layer overlapping design. This mechanism has a diameter adjusting ratio of 3.3, which enables effective anchoring and flexible crawling of the robot.

The principle of anisotropic friction-based vibrational crawling generally relies on the vibration of the actuator to exert momentum and the addition of tilted bristles to obtain anisotropic friction; the robot has a tendency to move in the direction with lower friction. Han et al. [18] developed a customized miniature vibration robot (Fiberbot) which uses a vibration motor as the actuator and a directional fiber pad to obtain directional friction. Song et al. [19] proposed a light-driven soft robot with awn feet. Structures that mimic the elastic awns of Setaria viridis were added to the three-layered composite film as the main body to generate anisotropic friction. Cao et al. [20] proposed a dielectric elastomer actuator (DEA)-driven reconfigurable crawling robot, demonstrating a fast-moving velocity of 0.9 BL/s. Zhan et al. [21] proposed a miniature vibration-driven planar locomotion robot which realized locomotion based on the internal oscillations of two parallel oscillators and the anisotropic friction from a blade-like support. Lu et al. [22] proposed a 3D printed anisotropic mobile robot, applying alternative magnetic fields to an electromagnet as an excitation source to control the vibration of the robot; anisotropic friction was generated using 3D printed anisotropic slender microfibers inspired by the structure of foxtail grass. Sheng et al. [23] designed a novel caterpillar-inspired pneumatic-driven soft crawling robot mainly composed of a pneumatic-driven bellows body, twelve anisotropic friction feet, and two end caps. The influence of the cross-section shapes of the robot feet on the performance of the robot was investigated and it was shown that the triangular feet can achieve superior movement efficiency, with a velocity of 1.05 cm/s (0.16 BL/s). Wu et al. [24] proposed a soft crawling robot with spike-inspired robot feet to obtain anisotropic friction and achieved a movement velocity of 0.275 mm/s when the twisted and coiled polymer actuator was powered at 24 V.

Although considerable progress has been made in research on crawling robots with the aforementioned driving mechanisms, there are limitations which affect their effective applications in real-world settings. In most of the existing designs, the two-anchor crawling principle requires a special anchoring mechanism, which results in a complex robot structure and manufacturing difficulties. The bristles used to obtain anisotropic friction are prone to contamination and wear in many cases. Meanwhile, the emerging vibro-impact crawling robots do not require complex anchoring mechanisms or tilted bristles to realize crawling, potentially reducing the complexity of manufacturing and further improving locomotion efficiency and stability [25]. A vibro-impact crawling robot mainly uses the directional impact force of the actuator to overcome the friction between the robot and the contact surface and to obtain the forward power needed to produce movement. Gu et al. [26] developed a nonlinear dynamic model of the vibro-impact capsule system and studied its dynamic responses under random environmental perturbation. Barenboim et al. [27] proposed a burrowing robot that can move and steer in granular media. The robot is driven by an internal vibro-impact mechanism and uses a rotary inclination head to realize steering. Liu et al. [28] proposed a millimeter scale vibro-impact capsule system with a length of 26 mm and a diameter of 11 mm for small intestinal endoscopy. A mathematical model was used to optimize its performance and a peak velocity of 14.4 mm/s was achieved. Duong et al. [29] studied the dynamic response of the vibro-impact capsule on the inclined track and a random slope. Zhang et al. [30] proposed a vibro-impact self-propelled capsule robot with azimuth control, which uses electromagnetic actuators for directional control and drives the robot to move forward or backward through internal vibro-impact. The experimental results showed a velocity of 2.66 mm/s on a piece of pig intestine tissue. Wu et al. [31] proposed a new bidirectional solenoid actuator for an active capsule robot. The robot mainly consists of an internal mass formed by a solenoidal coil wrapped around the iron core and two permanent magnets at the two ends of the capsule body. The robot can be driven by the internal impact force to achieve both forward and backward motion.

Although the research on vibro-impact robots has made great progress, the existing vibro-impact robots are mainly driven by servomotors or spiral coils to produce impact forces. These rigid actuators severely limit the adaptability of the robot in unstructured environments, and can potentially result in accidents during human–robot interactions. Compared with traditional rigid actuators, the novel soft robots rely on the stimulus response of soft materials to obtain the driving motion, and hence can better adapt to the complex environment and achieve safe human–robot interactions [32,33,34]. Dielectric elastomer actuators are an emerging type of soft actuator that can respond to electric field stimulation [35,36]. DEAs have the characteristics of large driving strain, high energy and power density, inherent flexibility, and low cost [37,38,39,40,41]. They have broad application prospects in soft robots [42], soft grippers [43,44], and wearable devices [45].

Despite the attractive features offered by the DEAs, to the best of the authors’ knowledge a DEA-driven vibro-impact robot has not yet been demonstrated, which could be due to the complex electromechanical coupling and vibro-impact nonlinear dynamics. Therefore, this paper explores the feasibility of using DEA as a soft vibro-impact source by proposing a novel type of DEA driven vibro-impact crawling robot. First, the dynamic characterization of the complex nonlinear system composed of an antagonistic DEA and an impact constraint is carried out. Then, the performance of the robot is analyzed in extensive experiments and the basic locomotion principle of vibro-impact crawling is studied in-depth. Finally, the effects of key actuation and design parameters, including the actuation voltage and frequency, constraint gap, and load mass, on the robot’s locomotion velocity are studied.

The rest of this paper is organized as follows. Section 2 introduces the design of the DEA and its dynamic performance characterization. The vibro-impact crawling robot is characterized in Section 3, which mainly introduces the design and working principle of the robot, the experimental setups, and characterization of the robot’s motion performance. Finally, the conclusions are provided in Section 4.

## 2. DEA Design and Characterization

### 2.1. DEA Design Overview

An ideal DEA consists of a dielectric elastomer (DE) membrane with two compliant electrodes covering its surface. Subject to an electric field, the attraction of electrostatic forces between the charges with opposite signs on the two electrodes causes the membrane to expand in plane and shrink in thickness. This paper adopts a double cone DEA (DCDEA) configuration as the motion generator. The DCDEA configuration has good output stability, a large stroke/force output, and simple manufacturing processes [46,47,48,49,50].

**Figure 1:**
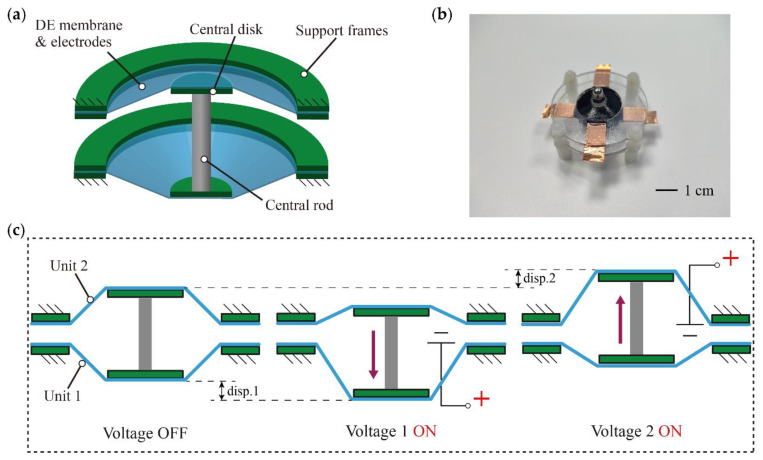
(**a**) Schematic diagram of the DCDEA; (**b**) Fabricated prototype of the DCDEA; (**c**) Actuation principle of the DCDEA.

The DCDEA consists of two identical conical DEA units connected by an intermediate rod as the end-effector. Each unit consists of a circular dielectric elastomer membrane, an annular support frame, and a central disk. The schematic illustration, fabricated prototype, and actuation principle of the DCDEA are shown in Figure 1a–c, respectively. It can be seen from Figure 1c that when the dielectric elastomer membrane is in the initial state without voltage applied, the two units have the same out-of-plane deformation. When voltage 1 is applied to unit 1, the DE membrane is deformed under Maxwell stress, causing the intermediate rod to move towards unit 1. However, when voltage 2 is applied to unit 2, it causes the intermediate rod to move towards unit 2. Thus, when voltages 1 and 2 are applied alternately to units 1 and 2, the end-effector oscillates back and forth.

### 2.2. DCDEA Fabrication and Experimental Setup

The fabrication process of the DCDEA is described below.

- A 100 µm silicone film (ELASTOSIL 2030, Wacker Chemie AG) was bonded to the acrylic support ring frame (20 mm inner diameter) using double-sided adhesive, and an 8 mm outer diameter acrylic center disk was bonded to the middle of the silicone film. The 20 mm ID was determined following the previous cone DEA studies [47,49], as a cone DEA with a smaller ID down to 1s mm becomes challenging in terms of fabrication.
- Custom-made carbon grease electrodes were applied evenly by a hand brush on both sides of the dielectric elastomer film.
- Two identical circular DE units were connected with nylon rods, while the outer frame was connected with shorter nylon rods such that the out-of-plane deformation of each film in the initial state was 2.5 mm. Note that the initial out-of-plane deformation of 2.5 mm results in a deformation-to-radius ratio of 0.25, which falls within the optimal ratio ranges reported in previous studies [39,46].

**Figure 2:**
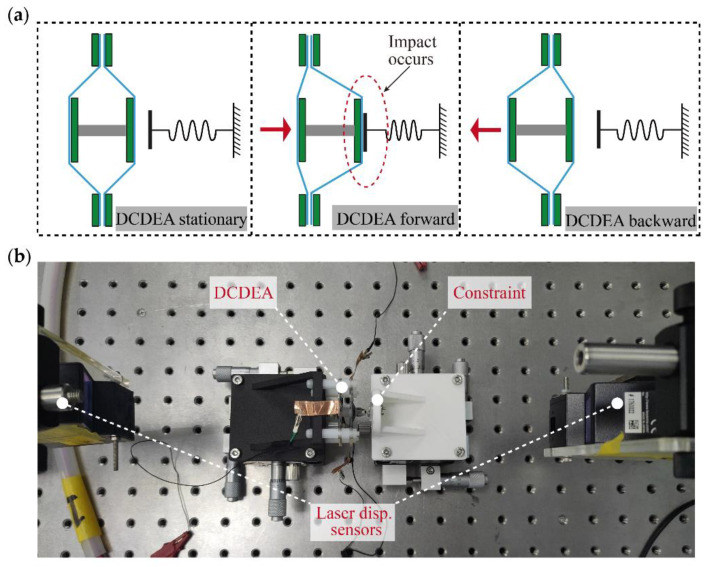
(**a**) Illustration of the DCDEA-based vibro-impact system and (**b**) experimental setup for the DCDEA dynamic test.

In this paper, the reciprocating oscillation principle of the DCDEA end-effector is adopted to realize a vibro-impact system by adding a constraint on one side of the movement direction of the DCDEA, as shown in Figure 2a. When the DCDEA moves forward, impacts between the constraint and the end-effector of the DCDEA occur. In order to investigate the effects of the added constraint on the nonlinear dynamics of the DCDEA, it is necessary to conduct dynamic characterization experiments on this system.

The experimental setup for the DCDEA dynamic test is shown in Figure 2b and is described as follows. The DCDEA and 2 mm thick acrylic constraint were each fixed on a separate displacement adjusting platform. The laser displacement sensor on the left (LK-G152 and LKGD500, Keyence) measured the deformation of the DCDEA at a sampling frequency of 20,000 Hz, and the laser displacement sensor on the right was used to determine the precise gap between the constraint and the DCDEA.

### 2.3. Analysis of DCDEA Dynamic Test Results

**Figure 3:**
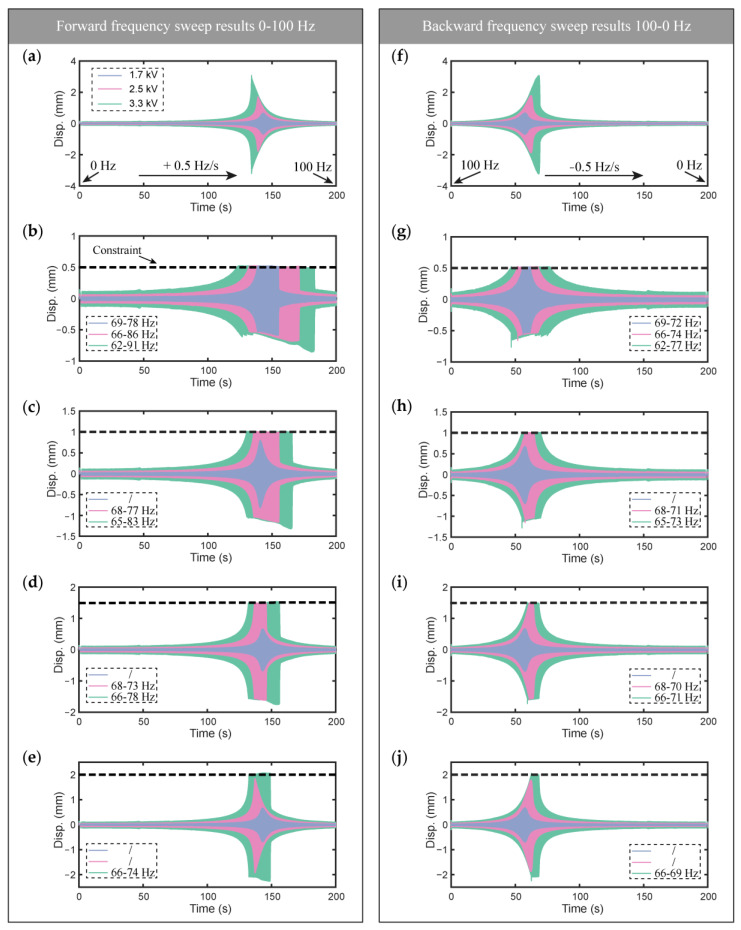
Forward and backward frequency sweep results of DCDEA and constraint with different gaps and under different actuation voltage amplitudes; (**a**) Forward frequency sweep result without constraint; (**b**) Forward frequency sweep result with constraint gap of 0.5 mm; (**c**) Forward frequency sweep result with constraint gap of 1 mm; (**d**) Forward frequency sweep result with constraint gap of 1.5 mm; (**e**) Forward frequency sweep result with constraint gap of 2.0 mm; (**f**) Backward frequency sweep result without constraint; (**g**) Backward frequency sweep result with constraint gap of 0.5 mm; (**h**) Backward frequency sweep result with constraint gap of 1 mm; (**i**) Backward frequency sweep result with constraint gap of 1.5 mm; (**j**) Backward frequency sweep result with constraint gap of 2.0 mm.

The forward frequency sweep (0–100 Hz within 200 s) and backward sweep (100–0 Hz within 200 s) voltage signals with amplitudes of 1.7, 2.5, and 3.3 kV were applied to the DCDEA. The voltage signal amplitude of 1.7, 2.5, and 3.3 kV corresponds to an electric field of 20, 30, and 40 V/μm, respectively, which falls within the safe operation range of the DE material (following [20,49]). The constraint gaps in these dynamic tests were determined as 0.5, 1, 1.5, and 2 mm, respectively. Figure 3 shows the measured frequency sweep results for the DCDEA. Among them, Figure 3a–e shows the forward frequency sweep results of the DCDEA under different constraint gaps and different voltage amplitudes, and Figure 3f–j shows the corresponding backward frequency sweep results. In addition, the frequency of impact with different actuation voltage amplitudes is marked in Figure 3b–e,g–j.

Figure 3a,f shows the free oscillation of DCDEA under voltage without constraint. It can be seen that, regardless of the forward or backward frequency sweeps, the increase in voltage amplitude causes an increase in the resonant amplitude and a decrease in the resonant frequency, which demonstrates the existence of nonlinearity in the DCDEA system. A comparison of Figure 3b–e and Figure 3g–j shows that the forward sweep has a wider impact frequency band than the backward sweep for the same constraint gap and voltage amplitude. It can be seen that regardless of whether the frequency sweep is forward or backward, increasing the constraint gap under the same actuation voltage results in a reduction in the impact frequency band. By comparing Figure 3a and Figure 3b–e or Figure 3f and Figure 3g–j, it can be seen that the occurrence of the constraint leads to an increase in the resonant frequency, with a smaller constraint gap leading to a more obvious deviation in the resonant frequency. This indicates that the system composed by the DCDEA and constraint has more complex nonlinearity, which demands further analysis. It is worth noting that the dynamic vibro-impact test of the DCDEA provides guidance for the design of the robot in the next step, that is, in locating the frequency band of the vibro-impact in the robot test, thus significantly reducing the workload during robot performance characterization.

In order to further study the effects of constraint on the DCDEA, in this paper we carried out a dynamic test of the vibro-impact system at a fixed frequency of 87 Hz, constant actuation voltage amplitude of 3.3 kV, and constraint gap of 0.5 mm, which shows the most severe distortion (that is, compared with the resonance frequency of DCDEA without constraint in Figure 3a, the deviation is the farthest) in the frequency response curves in Figure 3b. It can be seen from Figure 3b that in the case of forward frequency sweep, impact occurs at 87 Hz. However, note from Figure 3g that no impact is observed at 87 Hz in the backward frequency sweep result, indicating that the strong nonlinearity in this system results in multiple stable solutions (high amplitude oscillation with impacts and low amplitude oscillation with no impact) at this actuation frequency. By directly applying an actuation voltage at 87 Hz to the DCDEA system, it can only realize the low amplitude oscillation with no impact. Therefore, the test adopted a combination of the forward frequency sweep signal (50–87 Hz) and a series of fixed frequency signals (at 87 Hz) to the actuation voltage in order to realize the high amplitude oscillation with impacts. This allows the response to follow the high amplitude branch as the actuation frequency increases and is maintained at high amplitude oscillation with impacts at 87 Hz for further studies.

**Figure 4:**
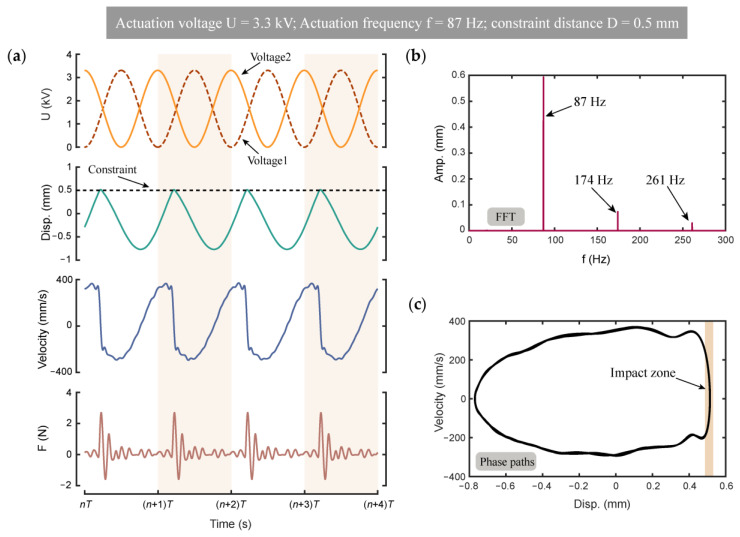
(**a**) DCDEA dynamic response results when the actuation voltage amplitude is 3.3 kV, the actuation frequency is 87 Hz, and the constraint gap is 0.5 mm; (**b**) Fast Fourier Transform (FFT) of the DCDEA oscillation displacement; (**c**) Phase paths diagram of the DCDEA.

The test results of four cycles with fixed frequency excitation at 87 Hz are shown in Figure 4. Figure 4a shows the two alternating sinusoidal voltages applied to DCDEA, the oscillation displacement of DCDEA, the velocity of DCDEA, and the impact force of DCDEA measured by a load cell (S/N 835827, FUTEK). Figure 4b shows the Fast Fourier Transform (FFT) of the DCDEA oscillation displacement. As can be seen from Figure 4a,b, under the constraint, DCDEA displacement is strongly affected and can be decomposed into multiple sinusoidal signals with different amplitudes and frequencies. Figure 4c shows the phase paths diagram of the DCDEA; the shaded part is the area where impact occurs. It is intuitive that when impact occurs, the constraint has a dramatic effect on the velocity of DCDEA, which rapidly changes from about 350 mm/s to −200 mm/s. As can be seen from Figure 4a, when the impact occurs, the impact force from the DCDEA on the constraint is about 3 N. The periodic impact force is applied to overcome the static friction and transfer kinetic energy to the robot, which is demonstrated in the following section.

## 3. Robot Design and Characterization

### 3.1. Overview of Robot Design

**Figure 5:**
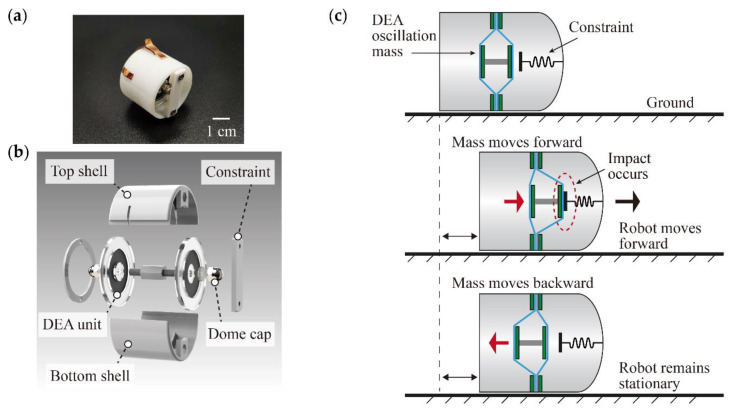
(**a**) Fabricated prototype of the vibro-impact crawling robot; (**b**) Key components of the robot; (**c**) Working principle of the robot.

The proposed vibro-impact robot mainly includes a cylindrical shell, a DCDEA, and an impact constraint. Its prototype is shown in Figure 5a. The annular support frame of the DCDEA is fixed to a 3D printed cylindrical shell and has rectangular holes for connecting high-voltage power sources. A steel cap is fixed to the end-effector of the DCDEA, which contacts the constraint fixed in front of the robot during impacts. Figure 5b shows the structural explosion diagram of the robot. The outer diameter of the prototype robot is 32 mm, the length is 30 mm, and the overall weight is 9.5 g. The key design parameters of the vibro-impact crawling robot are summarized in Table 1.

The working principle of the designed vibro-impact crawling robot is depicted in Figure 5c. In the initial state, the robot is stationary. When anti-phase sinusoidal AC voltages are applied to the two units of the DCDEA, the DCDEA drives the end-effector to produce reciprocating oscillation motion. When the end-effector moves forward and beyond the constraint gap D, the steel cap and the constraint fixed on the robot impact and generate an impact force. When the impact force is greater than the static friction force between the robot and the ground, the robot moves forward. When the end-effector moves backward, the force exerted on the robot is insufficient to overcome the static friction force, and the robot remains stationary. Therefore, the reciprocating oscillating motion of the DCDEA leads to the continuous forward motion of the robot.

### 3.2. Experimental Setup

**Figure 6:**
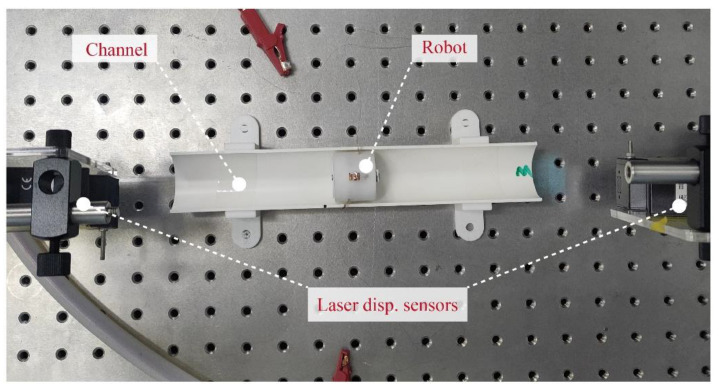
Experimental setup of the robot performance test.

The experimental setup of the robot performance test is shown in Figure 6. A PVC pipe with an inner diameter of 36 mm was fixed on the test bench. Anti-phase sinusoidal AC voltages were applied to the DCDEA with a fixed amplitude, frequency, and period of 3 s, which drove the robot to crawl in the pipe. The laser displacement sensor on the left measured the displacement of the DCDEA end-effector at a sampling frequency of 20,000 Hz. The laser displacement sensor on the right was used to measure the displacement of the robot. The test was repeated five times for each voltage amplitude and frequency combination to eliminate random errors.

### 3.3. Performance Characterization

#### 3.3.1. Effects of Actuation Voltage

**Figure 7:**
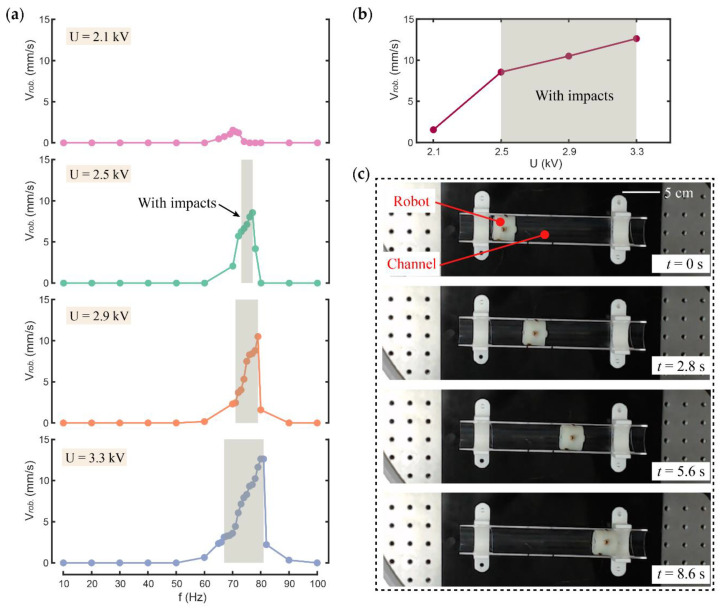
(**a**) Velocity of the robot at different actuation voltage amplitudes and frequencies; (**b**) Maximum velocity of the robot at different actuation voltage amplitudes; (**c**) Snap-shots of robot motion.

In order to investigate the effects of actuation voltage amplitude and frequency on the velocity of the robot, voltage amplitudes of 2.1, 2.5, 2.9, and 3.3 kV and an actuation frequency of 10–100 Hz under the condition of constraint gap D = 0.6 mm were tested. The experimental results are shown in Figure 7, where the gray shaded areas represent the actuation frequency with the occurrence of impacts.

Figure 7a shows the velocity of the robot under different actuation voltage amplitudes and different frequencies. It can be seen that the robot demonstrates a low velocity or even remains stationary at actuation frequencies with no impact occurring. At the actuation frequencies with impacts, the robot shows a substantially faster forward velocity, which proves the effectiveness of the design. Figure 7b shows the change in the maximum velocity of the robot under different actuation voltage amplitudes. As can be seen from Figure 7a,b, with the increase in voltage amplitude, the impact area and peak velocity of the robot increase simultaneously. Under the actuation voltage of 3.5 kV and 80 Hz, the maximum velocity of the robot is 12.6 mm/s, i.e., 0.42 BL/s. Figure 7c is a series of snap-shots of the robot actuated with voltages of 3.5 kV and 80 Hz.

**Figure 8:**
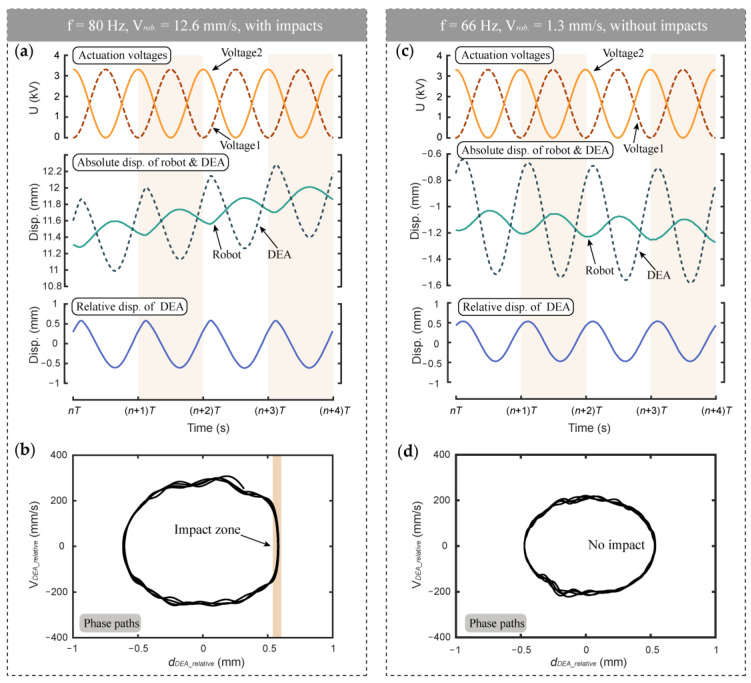
(**a**) Time series of the measured displacements and (**b**) phase paths of the relative DCDEA oscillation at 80 Hz with impacts; (**c**) Time series of the measured displacements and (**d**) phase paths of the relative DCDEA oscillation at 66 Hz without impacts.

In order to further study the actuation mechanism of the vibro-impact robot, the movement details of the DCDEA and the robot when the voltage amplitude is 3.3 kV and the actuation frequency is 80 Hz and 66 Hz are shown in Figure 8. It can be seen that the robot reaches its peak speed at the actuation voltage of 3.5 kV and 80 Hz. It is worth noting that this frequency is less than 87 Hz of the DCDEA when the constraint gap is 0.5 mm in Section 2.3. This is because at 87 Hz, although the amplitude of the DCDEA is at its maximum, its stability is extremely weak. Therefore, when the actuation frequency of 87 Hz is directly applied, high-amplitude oscillation cannot be achieved to generate impact during robot locomotion. For this reason, the actuation frequency of 80 Hz where the robot achieves the maximum speed is used here.

Figure 8a,b shows the time series information and phase paths of vibro-impact, respectively, when the robot moves at the maximum velocity of 12.6 mm/s at the 80 Hz actuation frequency. Figure 8a shows that the absolute displacements of the robot and the DCDEA indicate that the robot oscillates back and forth in one cycle and shows a net forward progression. The displacement of the DCDEA relative to the robot shows that the DCDEA oscillates periodically with respect to the robot. It can be seen from Figure 8b that the impact occurs at D = ~0.6 mm, which is consistent with the above settings. When the impact occurs, the direction of movement of the DCDEA changes rapidly and transfers energy to the robot to drive it forward.

As a comparison, the time series information and phase paths of the robot at a velocity of 1.3 mm/s without impacts are shown in Figure 8c,d where the voltage amplitude remains unchanged and the actuation frequency is 66 Hz. As can be seen from Figure 8c, due to the inertia of the DCDEA, the robot oscillates bidirectionally instead of staying stationary, while its net forward displacement is obviously smaller than is the case with impacts (shown in Figure 8a). It is worth noting that while the amplitudes of the DCDEA are close in the two cases, the amplitude in Figure 8d is slightly lower than the impact gap D, which is unable to trigger impacts, resulting in a very low robot velocity. Therefore, it can be seen from Figure 8 that the use of the vibro-impact mechanism is indeed able to achieve a significant improvement in the robot’s locomotion performance.

#### 3.3.2. Effects of Constraint Gap (D)

**Figure 9:**
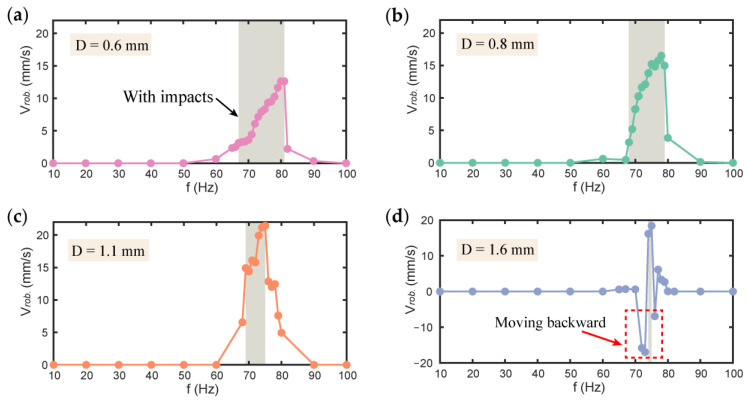
(**a**) The velocity of the robot with constraint gap of 0.6 mm; (**b**) The velocity of the robot with constraint gap of 0.8 mm; (**c**) The velocity of the robot with constraint gap of 1.1 mm; (**d**) The velocity of the robot with constraint gap of 1.6 mm.

In order to investigate the effects of constraint gap (D) on the velocity of the robot, robot prototypes with different constraint gaps from 0.6–1.6 mm were tested with the actuation frequency varied from 10–100 Hz and amplitude fixed at 3.3 kV. The test results are shown in Figure 9, where the gray shaded areas represent the actuation frequency where impact occurs on the robot.

Figure 9a–d shows the velocity of the robot with the constraint gaps of 0.6, 0.8, 1.1, and 1.6 mm, respectively. It can be seen that with the increase of constraint gap, the frequency band of the impact region decreases, which is demonstrated in Figure 3 as well. However, the maximum velocity of the robot increases first from 12.6 mm/s at D = 0.6 mm to 21.4 mm/s at D = 1.1 mm, then decreases slightly to 18.4 mm/s at D = 1.6 mm. It is worth noting that our robot has a peak velocity of 0.71 BL/s. Compared with the vibro-impact robots reported in the literature [28,30,51,52,53], the proposed robot has a clear advantage in its maximum velocity, which offers potential advantages in applications such as disaster search and rescue. In addition, it is noteworthy that when D = 1.6 mm, the robot exhibits noticeable backward locomotion near the impact frequency band, as highlighted in the red box in Figure 9d. At 73 Hz, the maximum backward velocity of the robot reaches 16.9 mm/s. This finding shows that by a simple tuning in the actuation frequency in the case of D = 1.6 mm we can actively control both the locomotion direction and the velocity.

#### 3.3.3. Effects of Load Mass (M)

**Figure 10:**
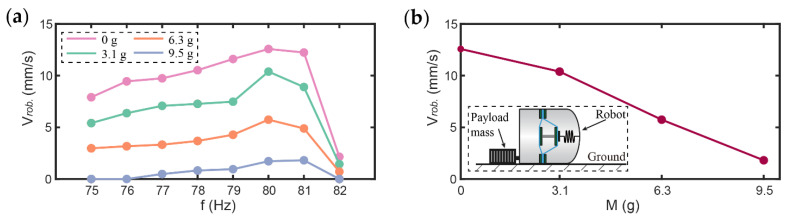
(**a**) The velocity of the robot with load mass of 0, 3.1, 6.3, and 9.5 g; (**b**) Effects of load mass on the velocity of the robot.

To investigate the effects of load mass (M) on the velocity of the robot, a different load mass up to 9.5 g was added to the robot while the voltage amplitude was fixed at 3.3 kV and a constant constraint gap D of 0.6 mm was adopted. To apply the load mass to the robot, cylindrical magnets with different masses are connected to the rear end of the robot for dragging; the specific schematic diagram is shown in the subfigure in Figure 10b. Note that based on the findings in Figure 7 and Figure 9,that is, that the peak velocity of the robot occurs near the upper end of the impact frequency band, the actuation frequency was varied from 75–82 Hz in this study. The test results are shown in Figure 10.

Figure 10a shows the variation of the robot’s velocity with the actuation frequency when the load mass is 0, 3.1, 6.3, and 9.5 g, respectively. Figure 10b shows the variation of the maximum forward velocity of the robot with the load mass. It can be seen that the velocity of the robot decreases approximately linearly with the increase in the load mass. However, the robot remains able to move at a velocity of 1.8 mm/s with a load mass of 9.5 g (equivalent to its own body weight), demonstrating a reliable load carrying capability.

### 3.4. Robot Demonstrations

To demonstrate the feasibility of practical applications of the proposed vibro-impact crawling robot, we show the motion of the robot in a curved pipe and several substrates with different friction coefficients which resemble the simplified forms of different practical real-world applications.

**Figure 11:**
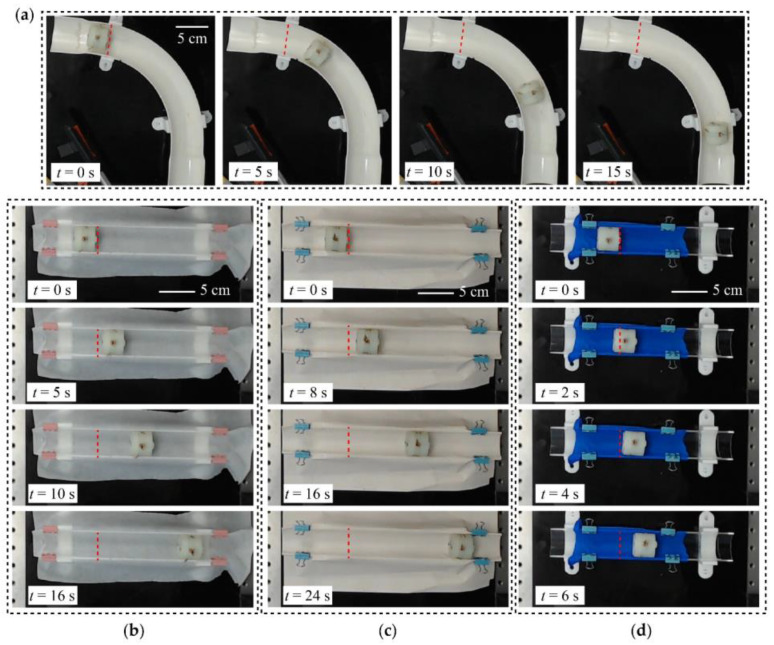
(**a**) Demonstration of the robot passing through a curved pipe; (**b**) Demonstration of the robot crawling through a non-sticking foil surface; (**c**) Demonstration of the robot crawling through a paper surface; (**d**) Demonstration of the robot crawling through a rubber surface.

In these demonstrations, the robot was driven by two anti-phase sinusoidal voltages of 80 Hz and 3.3 kV amplitude. Figure 11a shows snap-shots of the robot crawling through a curved pipe with a curvature radius of ~15 cm, and Figure 11b–d shows snap-shots of the robot crawling through a non-sticking foil surface, a paper surface, and a rubber surface, respectively. As can be seen from Figure 11, the designed vibro-impact crawling robot is capable of traveling on various types of substrates with different friction coefficients, which demonstrates the high degree of adaptability and potential for practical application.

### 3.5. Results Discussion

In this subsection, the vibro-impact robot proposed in this paper is compared with existing vibro-impact robots, as summarized in Table 2. As can be seen from Table 2, compared with the vibro-impact robots reported in the literature, the proposed robot has a clear advantage in maximum velocity (BL/s). The peak velocity of the proposed robot is about 30% faster than the fastest existing vibro-impact robot, meaning that it has an advantage in applications such as disaster search and rescue. DEAs can be powered by a supply unit which weighs less than 1 g [54], and the actuator itself can be as a light as tens of milligrams [55], which can be adopted to realize extremely light and portable vibro-impact crawling robots for surveillance or medical robotics. It is noteworthy that DEAs down to a millimeter scale [56] have been proposed, which potentially enable DEA-driven crawling robots to operate in constrained environments such as aircraft pipeline inspections.

## 4. Conclusions

A novel vibro-impact crawling robot driven by DCDEA was proposed in this paper. First, the dynamic responses of the system composed of the DCDEA and impact constraint were characterized extensively in experiments. Based on these, the performance of the robot was studied, the basic principle of vibro-impact crawling was analyzed, and the effects of several key actuation and design parameters (including actuation voltage amplitude and frequency, constraint gap, and load mass) on the performance of the robot were characterized.

The main findings of this paper are summarized as follows:

1. An actuation system composed of an impact constraint and a DCDEA produces complex nonlinear phenomena. A smaller constraint gap leads to stronger distortions in the frequency response curve of the DCDEA by causing a substantial increase in its resonant frequency.
2. In comparing the performance of the robot with and without vibro-impacts, we showed by experiments that the vibro-impact has a decisive effect on the peak velocity of the robot, confirming the effectiveness of the design.
3. The designed robot can realize bidirectional locomotion (both forward and backward) by a simple adjustment of the determinant parameters (i.e., actuation frequency and constraint gap). The robot achieved a forward velocity of up to 21.4 mm/s (0.71 BL/s) and a backward velocity of 16.9 mm/s. The robot showed good load capability, moving a load of 9.5 g (equivalent to its own body weight) at a velocity of 1.8 mm/s.

To the best of the authors’ knowledge, this paper presents the first vibro-impact crawling robot driven by a soft actuator consisting of DEAs. The lower stiffness of the DEA (close to human tissues) compared with rigid motors and electromagnetic coils can potentially result in safer human–robot interactions, and has promise in capsule endoscopes. However, it is worth noting that the robot shell in this paper was 3D printed for the sake of rapid prototyping and characterization. Future work should further explore the feasibility of realizing a fully soft vibro-impact robot, e.g., by adopting a PDMS-cast shell. Human gastrointestinal tracks are typically covered with mucus, which differs from the dry friction conditions tested in this paper. Hence, future work should investigate the feasibility and principle of our vibro-impact robot in viscous environments [57,58]. The performance of the proposed robot (maximum velocity and load capacity) can be improved by stacking more layers of DE membranes, using better performing DE and electrode materials, etc., all of which will be explored in our future research. The vibro-impact crawling robot driven by DCDEA proposed in this paper has potential applications in industrial pipeline inspections, capsule endoscopes, and disaster rescue.

## Figures and Tables

**Table 1:** Key design parameters of the vibro-impact crawling robot.

Design Parameters	Values	Units
Length	30	mm
Outer diameter	32	mm
Total weight	9.5	9.5
DCDEA moving mass	1.7	g
DCDEA electrode diameter	20	mm
DCDEA out-of-plane deformation per unit	2.5	mm
DCDEA membrane thickness	100	µm
Impact gap	0.6–1.6	mm

**Table 2:** Comparison of the proposed robot and existing vibro-impact crawling robots.

Reference	Dimensions (mm)	Actuation Source	Weigh t(g)	Peak Velocity (BL/s)
[51]	OD 80	Linear DC servomotor	500	~0.1
[28]	26 × OD 11	An internal magnet and an external coil	3.47	0.55
[52]	/	Electro-dynamical shaker	2336	/
[30]	34 × OD 15	Four internal coils and an internal magnet	5.38	0.16
[53]	26 × OD 11	An internal magnet and two external coils	3.47	0.13
Current work	30 × OD 32	DCDEA	9.5	0.71
